# Real-Time Liquid Rate and Water Cut Prediction From the Electrical Submersible Pump Sensors Data Using Machine-Learning Algorithms

**DOI:** 10.1021/acsomega.2c07609

**Published:** 2023-03-30

**Authors:** Ramez Abdalla, Waleed Al-Hakimi, Nelson Perozo, Philip Jaeger

**Affiliations:** Institute of Subsurface Energy Systems, Clausthal University of Technology, Agricolastrasse 10, 38678 Clausthal-Zellerfeld, Germany

## Abstract

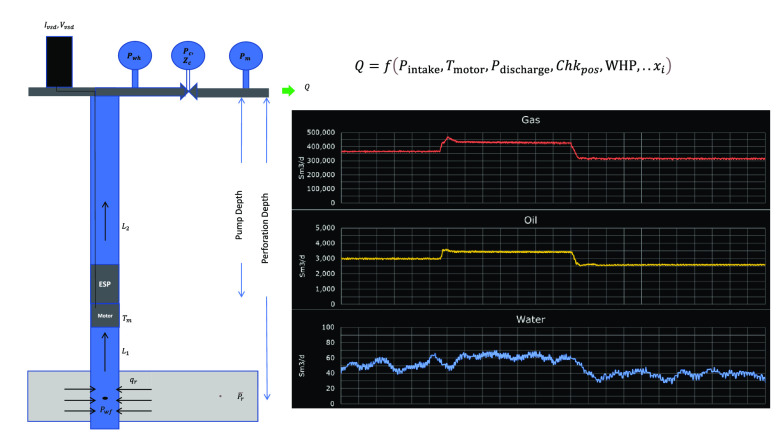

This study presents a novel data-driven approach for calculating multiphase flow rates in electrical submersible pumped wells. Traditional methods for estimating flow rates at test separators fail to identify production trends and require additional costs for maintenance. In response, virtual flow metering (VFM) has emerged as an attractive research area in the oil and gas industry. This study introduces a robust workflow utilizing symbolic regression, extreme gradient boosted trees, and a deep learning model that includes a pipeline of convolutional neural network (CNN) layers and long short-term memory algorithm (LSTM) layers to predict liquid rate and water cut in real time based on pump sensors’ data. The novelty of this approach lies in offering a cost-effective and accurate alternative to the usage of multiphase physical flow meters and production testing. Additionally, the study provides insights into the potential of data-driven methods for VFM in electrical submersible pumped wells, highlighting the effectiveness of the proposed approach. Overall, this study contributes to the field by introducing a new, data-driven method for accurately predicting multiphase flow rates in real time, thereby providing a valuable tool for monitoring and optimizing production processes in the oil and gas industry.

## Introduction

Flow rate estimation is crucial for the production monitoring of wells.^1^ Production testing is a commonly used method for estimating flow rates by diverting the flow of a particular well to a test separator. However, production testing has several drawbacks such as lack of sufficient testing time, inadequate resolution, technical disadvantages, and high costs. In recent years, multiphase flowmeters (MPFMs) have emerged as an alternative to production testing. MPFMs estimate well multiphase flow rates indirectly by measuring properties of the fluid, such as density, phase velocities, and fraction of these phases inside the device.^3−6^ The most important advantage of MPFMs is that they are able to estimate the flow rate of each phase without having to separate them.

However, MPFMs are expensive, and their accuracy declines beyond a certain operating range.^3,7^ In addition, the meters may deteriorate from sand erosion or partial obstruction, affecting measurement accuracy. To overcome these difficulties, virtual flow metering (VFM) presents an efficient and economic approach. The purpose of VFM is to gather field data that is immediately available and implement it in a numerical model in order to calculate flow rates. VFM systems do not require the installation of any additional hardware, reducing capital and operating expenses. VFM systems are capable of estimating flow rates in real time and detecting changes of flow conditions accordingly.

In conclusion, the use of virtual flow metering (VFM) has the potential to become a critical tool for accurately estimating flow rates in the oil and gas industry. However, to implement this tool, detailed simulations of the production system are required. These simulations must encompass the near well bore area, wells, pipelines, and production chokes. These models are then combined with observations such as pressure and temperature. Then, optimization algorithms are employed to minimize the discrepancies between the model predictions and the actual measurements. The production system can be represented as a whole or divided into submodels, with most commercial systems utilizing first-principles models. Alternatively, data-driven VFM techniques have emerged as a promising alternative in recent years. This approach involves mathematically adapting field data to the production system’s physical parameters without explicitly describing them.

The purpose of data-driven VFM is to gather field data that are immediately available and implement it in a numerical model in order to calculate flow rates. Typically, the measurement data consist of (i) pump intake pressure and discharge pressure (*P*_*intake*_ and *P*_*discharge*_); (ii) wellhead pressure and temperature (WHP and WHT); (iii) choke opening (*C*_*op*_); (iv) pump vibration in axial and radial directions (*V*_*x*_ and *V*_*y*_); and (v) variable-speed drive current (*I*_*VSD*_).

VFM systems in general do not require the installation of any additional hardware, in contrast to production testing and MPFMs. By this means, it reduces the field development’s capital and operating expenses. At the same time, VFM systems are capable of estimating the flow rates in real time and detecting changes in flow conditions accordingly. Another important advantage compared to production testing is that VFM shows the production trend of the well. Moreover, VFM can be used as a standalone solution or in combination with an MPFM as a backup system so that it can use the information from an MPFM to further improve the estimation of flow rates.

In this paper, a contribution in the area of data-driven virtual flow metering on wells produced with electrical submersible pumps is introduced. Real data from a field is analyzed and handled through multiple machine learning algorithms in order to predict flow rate and water cut. In the following sections, the concepts of mechanistic and data-driven modeling are presented, and their implementation on electrical submersible pumps is specifically reviewed. Afterward, a data-driven modeling methodology based on a real ESP data set is developed. This methodology includes exploratory data analysis and experiments. Model evaluation and testing are carried out against a testing data set that is not used during the training stage.

## Literature Study

One of the most common artificial lift techniques is the employment of an electric submersible pump (ESP) to produce oil when natural production is impossible due to a variety of reasons, including low bottomhole pressure, liquid loading, and the presence of heavy oil. This technology is also the preferred choice for high production rates. There have been several attempts to develop a first-principles model that describes ESP operation, due to its widespread use. The main concept underlying virtual flow metering in ESP is to link the pump pressure increase, which is the difference between the intake pressure and the discharge pressure, with the flow and the pump speed while also considering the liquid percentage and gas volume.^9^ Earlier attempts for flow rate computation and prediction are displayed in Table 1, which shows the authors and the summary of their work.

**Table 1:** Literature Study

Author	Model summary
Camilleri et al., 2016a; Camilleri et al., 2016b; Camilleri et al., 2015; Camilleri et al., 2017; Camilleri and Zhou, 2011	In these papers, ESP models with different modifications are discussed, as well as field case studies where ESP first principles models serve as virtual flowmeters. A hybrid method has been used to measure the flow rate without needing a test separator or multiphase flowmeter. The principle states that the pump absorbs an amount of power that is generated by the motor. The drop in pressure in the tubing provides measurements of the average density of the fluid, which is then converted to a water cut. To obtain a reference for validating the calculation, a comparison has been carried out between the calculated rate and the measured rate on a shale oil well equipped with an ESP.^9−13^
Haouche et al., 2012b; Haouche et al., 2012a	The VFM model is a combination of three main units: the reservoir unit, the electrical submersible pump unit, and the production tubing unit. A density correction factor is used to take into account the effect of gas on the operational performance of the submersible pump.^14,15^
Awaid, 2014	Pattern recognition analysis was implemented to predict failures faster, enabling quicker reactions and optimal solutions. The input parameters used were flow rate, wellhead pressure, speed drive current, discharge pressure (*P*_*discharge*_), intake pressure (*P*_*intake*_), and motor temperature. By comparing real-time patterns of surface and downhole data with simple physical correlations, this study was able to anticipate well and reservoir ESP performance reliably.^16^
Binder et al., 2015	They considered moving horizon estimator for flow rate estimation in a well with an ESP. As input parameters they used bottomhole and pump data, including pump pressure sensors. The method showed an accurate performance and was suggested to be used for industrial applications.^17^
Zhu et al., 2016	In this research, singular spectrum analysis (SSA) was used on a raw production data set without any preprocessing or transformation of the original series. The group investigated the decomposition of the original series into a summation of the principal independent and explainable components such as slowly varying trends, cycling components, and random noise.^18^
Diaz de Bonilla, 2019	This study used analytical formulas and numerical computational fluid dynamics (CFD) simulations to reduce the ESP impeller erosion rate in order to improve the pump’s operating lifespan. The impeller’s geometrical features, rotational velocity, physical properties of the eroded material’s solid particle like density, and mass flow rates of the liquid and solid phases are used as input parameters. This method showed that the lower the particle velocity, the slower is the erosion rate. By increasing the solids density, the erosion in the pump’s impellers leading to ESP failures slows down.^19^
Agrawal, 2019	This study presented a group of diagnostic methods and tools that have been developed to analyze and understand production performance degradation in wells lifted by ESPs in the Mangala field with highly abundant polymer floods.^20^
Krikunov, 2019	A numeric optimization model was created to suggest multiwell operating modes, and a hybrid physical–ML prediction model was created and trained. With no additional capital investment needed, this technology, which offers the ESP optimal control solution, might increase the effectiveness of oil extraction by increasing oil well production rates by 1.5%.^21^
Zhu, 2019	This study utilized a novel mechanistic model to forecast oil–water emulsion rheology and predict how it will affect ESP boosting pressure.^22^
Khabibullin, 2020	This study describes the methodology “testing of an algorithm” of ESP failure prediction, which is developed depending on field data to reach the best directions for further improvement of the developed methodology that was used to avoid major negative consequences of ESP equipment failure.^23^
Bermudez, 2021	This study used real-life application of a machine learning method to predict imminent and future failures, extending pump run life and maximizing the production of electrical submersible pumps (ESPs). Failure prediction index (FPI), remaining run life (RRL), and virtual flow metering (VFM) are some of the machine learning models that were implemented in this paper.^24^
Barrios and Lissett, 2021	Pump and motor performance were estimated under two crucial factors, high viscosity and two-phase flow inside the ESP, through analysis and data comparison with field operation. By using this approach, the pump’s performance may be predicted with a thorough study of the physical layout, fluid flow routes, ESP power delivery system, pump and motor performance, and caisson separation characteristics.^25^
Sabaa et al., 2022	This study aims to develop artificial neural network models to predict flow rates of ESP artificially lifted wells. Each data set included measurements for wellhead parameters, fluid properties, ESP downhole sensor measurements, and variable speed drive (VSD) sensor parameters. The models consisted of four separate neural networks to predict oil, water, gas, and liquid flow rates.^26^

### Key Findings from the Relevant Literature

The literature study indicates that there is a growing interest in developing virtual flowmeter models for ESP wells using data-driven approaches. The reason behind this is that data-driven models offer a more efficient and accurate solution for real-time monitoring and control of production rates. However, the use of data-driven models in the ESP well industry is still in its early stages, as most of the previous work focuses on the mechanistic models within specific operating limits.^19,21,25,27^ Consequently, there is a need for further investigation and research to develop stable and robust models that be can generalized to various operating conditions.

One of the major challenges in developing stable and accurate data-driven models is the availability of high-quality training data. In many cases, the available data may be incomplete, noisy, or biased, which can affect the performance of the models. Therefore, it is crucial to preprocess the data and identify the relevant features that can best represent the system’s behavior. Hence, we propose a detailed exploratory data analysis in our methodology to study the sensor data from the ESP pumps before modeling.

Additionally, it is essential to use appropriate machine learning and deep learning algorithms that can effectively capture the underlying patterns and relationships in the data. This leads to another challenge in developing data-driven models, which is the lack of transparency and interpretability of some algorithms. From the data-driven applications studied in the literature,^24,26^ we have concluded that their workflow starts with the usage of more complex black-box algorithms without applying more interpretable machine learning algorithms. Therefore, the proposed methodology comprises three modeling steps: symbolic regression, XGBoosting, and deep learning. Specifically, the modeling stage initiates with the most interpretable application capable of obtaining functions that describe the system. Subsequently, we employ XGBoosting, which is a tree-based approach. Lastly, we employ deep learning algorithms and compare the findings to actual liquid rate and BS&W measurements.

Moreover, the literature study emphasizes the importance of assessing the predictability of data-driven models using an independent testing data set. This practice is vital to guarantee the models’ generalization ability to new operating conditions and their robust predictive performance. Nevertheless, previous research in the literature failed to conduct such an evaluation and solely relied on training and testing the models on the same data set. This methodology can result in overestimating the models’ performance and lead to suboptimal performance when implemented on new data.

In summary, the literature study suggests the existence of significant potential for developing stable and accurate data-driven models for virtual flow metering of electric submersible pump (ESP) wells. However, additional research is necessary to overcome the challenges associated with data quality, algorithm selection, and model interpretability. Furthermore, it is crucial to evaluate the models’ predictability using an independent testing data set to ensure robust performance in real-world applications, which was carried out in the current work.

### Comprehensive Workflow for Building Virtual Flow Metering Models of Electrical Submersible Pumps

**Figure 1:**
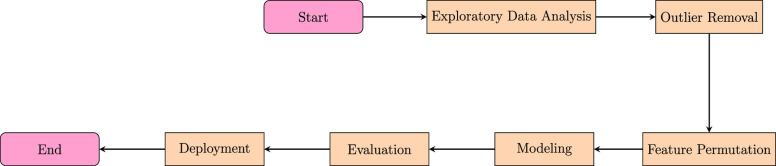
Workflow diagram.

In this section, we describe a comprehensive workflow for developing virtual flow metering (VFM) models in the oil and gas industry. Figure 1 presents the various steps involved in this workflow. To evaluate the performance of the VFM models, we use standard metrics and compare the results to select the most accurate model. Our workflow can be applied to other oil and gas applications, highlighting the potential of VFM to transform the industry.

1. Exploratory data analysis: The raw data from ESP sensors is collected and preprocessed to remove any missing values or anomalies. Hence, the data is analyzed to identify patterns, trends, and correlations between the variables using techniques such as scatter plots, correlation analysis, and clustering analysis.
2. Outlier removal: Any outliers or data points that may be erroneous or generated by measurement errors or sensor malfunctions are identified and removed from the data set to ensure that the model is trained on high-quality data.
3. Feature permutation: The most important features that influence the ESP performance and flow rates are identified. Therefore, feature permutation tests are performed to identify the top-performing features that provide the most accurate predictions of the flow rates.
4. Modeling:
  - Symbolic regression is used to develop a mathematical model that can accurately predict the ESP flow rates based on the most important features identified in the previous step.
  - XGBoosting is used to develop an ensemble model by combining multiple decision trees.
  - Deep learning is used to develop a neural network that can learn the complex patterns and relationships in the data.
5. Evaluation: The evaluation of the liquid rate, basic sediments, and water models is performed using commonly used performance metrics such as mean absolute error (MAE), mean squared error (MSE), and coefficient of determination (R-squared). These metrics are utilized to assess the accuracy and reliability of the models in predicting the desired outputs based on the input data.
6. Deployment: The selected model is deployed to provide real-time predictions of the ESP flow rates based on the sensor data, and the results are compared against real reported values.

This workflow provides a systematic approach to applying VFM on ESP and has been shown to provide accurate predictions of flow rates. The results obtained from this approach can be used to optimize the performance of ESPs and improve the efficiency of oil and gas production.

### Exploratory Data Analysis

Exploratory data analysis (EDA) helps to reveal hidden patterns, correlations, and anomalies using various tools such as visualizations (scatter and box plots, histograms, etc.), dimensionality reduction techniques, and sometimes unsupervised algorithms. Based on Hair et al. (2013),^28−31^ this project’s exploratory data analysis includes univariate study that focuses on the dependent variables flow rate and water cut. Then, a multivariate study is likewise introduced to focus on the relationship between dependent and independent variables. Clustering and dimensionality reductions are also studied in this step to investigate the categorical variables. Finally, basic cleaning is implemented to handle the missing data and outliers.

The reported signals coming from ESP pumped wells are categorized into 4 different groups. The first group is the wellhead data, which include choke opening (%), wellhead pressure (psig), flow line pressure (FLP) (psig), casing pressure (psig), and wellhead temperature (°F). The second group is the electrical data, which include frequency (Hz), input voltage of the three phases, variable speed drive (VSD), and output current of the three phases (amps). The third group is the operational downhole data, which include pump intake pressure (psia), pump discharge pressure (psia), pump intake temperature *T*_*i*_ (°C), motor temperature *T*_*m*_ (°C), vibration in 2D (*V*_*x*_, *V*_*y*_), and differential pressure (psia). The fourth and last group would be MPFM data, which are water cut in percent and produced fluid rate in BPD.

Before conducting exploratory data analysis (EDA) on a data set, it is important to first examine some basic statistical parameters of the data. These parameters can provide insights into the central tendency, spread, and shape of the data distribution. Commonly used statistical parameters for numerical data include the mean, median, mode, range, standard deviation, etc. Table 2 shows the statistical parameters for the sensor data and target outputs.

**Table 2:** Statistical Parameters for the Collected Data

	Choke (%)	WHP (psig)	FLP (psig)	Cas. P.	WHT (°F)	Freq Hz	Input volt	
count	33334.00	33286.00	32938.00	34026.00	32390.00	32780.00	31206.00	
mean	47.87	251.04	69.18	8.39	137.21	43.57	400.62	
std	35.79	141.16	43.97	21.00	20.74	7.52	35.01	
min	0.00	0.00	0.00	0.00	0.00	0.00	39.00	
25%	27.00	142.00	48.00	0.00	127.00	39.00	396.00	
50%	39.00	238.00	54.00	0.00	141.00	42.00	400.00	
75%	52.00	350.00	78.00	0.00	152.00	47.00	403.00	
max	192.00	755.00	415.00	100.00	508.00	60.00	4110.00	

	VSD (amps)	DH (amps)	*P*_*i*_ (psia)	*P*_*d*_ (psia)	*T*_*i*_ (°C)	*T*_*m*_ (°C)	*V*_*x*_	*V*_*y*_
count	32736.00	30408.00	34622.00	34622.00	32294.00	32288.00	32283.00	32168.00
mean	280.28	46.51	1917.84	2402.55	71.88	85.45	0.14	0.15
std	180.43	21.16	308.18	360.29	9.24	16.60	0.10	0.10
min	0.00	0.00	0.00	0.00	0.00	0.00	0.00	0.00
25%	182.00	31.00	1768.00	2260.00	67.84	76.40	0.08	0.10
50%	241.00	43.00	1981.00	2433.00	73.80	84.93	0.12	0.14
75%	302.00	55.00	2093.00	2595.50	77.59	90.20	0.18	0.19
max	1102.00	272.00	4150.00	4149.00	654.10	927.00	1.40	2.08

	Rate (BPD)	BS&W %						
count	31827.00	32049.00						
mean	5472.70	15.57						
std	5742.02	19.44						
min	0.00	0.00						
25%	1879.00	0.05						
50%	3523.00	5.70						
75%	6369.50	27.50						
max	27795.00	100.00						

From Table 2, it is seen that the mean is higher than the median for the liquid rate and BS&W, which indicates that there are large values in both signals that are pulling the mean upward, resulting in a skewed distribution. The median represents the middle value of the signal, so it is less affected by extreme values compared to the mean. Therefore, when the mean is significantly higher than the median, it suggests that both signals have a positive skewness and the distribution of values is not symmetrical. However, it is important to note that this may not always be the case, and therefore, it is necessary to analyze the data further to gain a better understanding of its distribution and characteristics.

**Figure 2:**
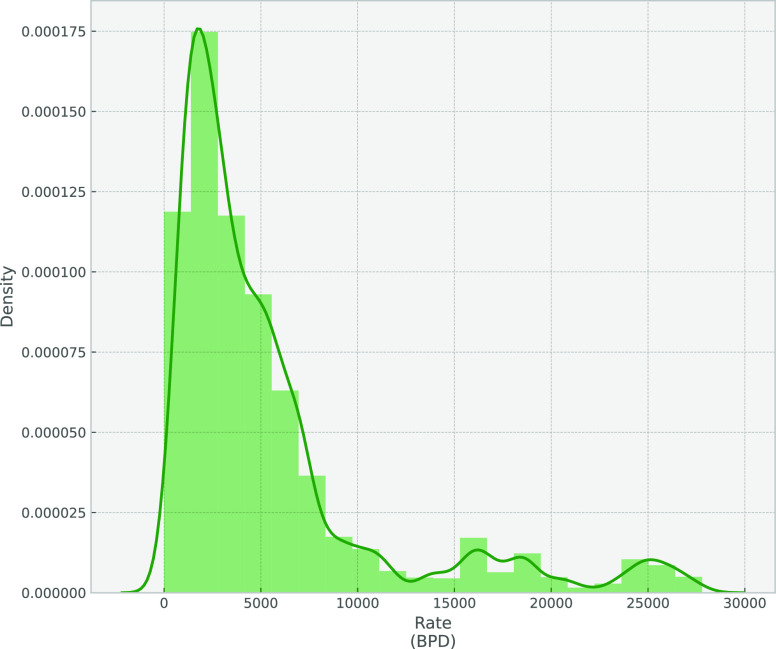
Density plot for fluid rate.

**Figure 3:**
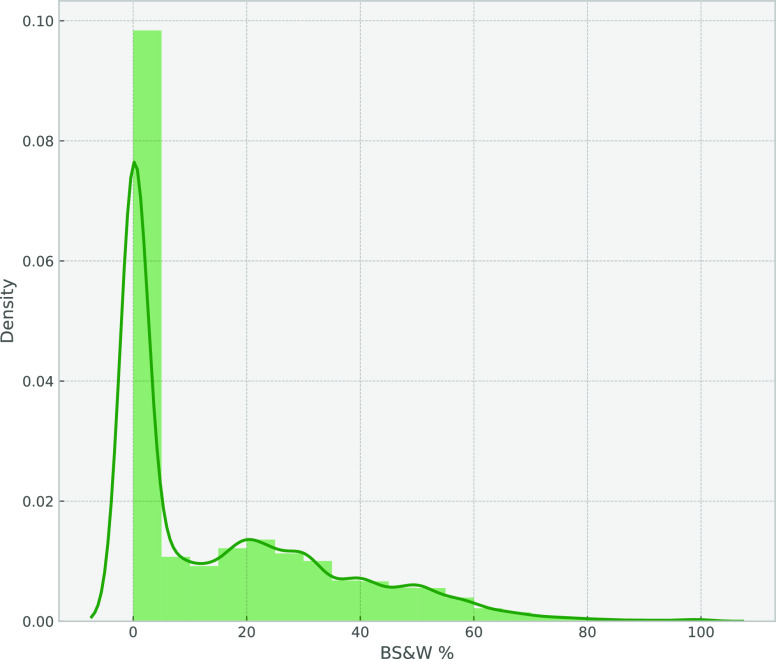
Density plot for water cut.

Proceeding with EDA, we start with the univariate study, in which the focus is first on the understanding of the dependent variables fluid rate and water cut. Figures 2 and 3 show the distribution plot for each signal.

It is obvious that the rates are skewed left and that some outliers lie above 15 000 BBL. The same happens for water cuts above 60%. Calculating the skewness and kurtosis for both signals shows that the fluid rate has a skewness of 2.09 and a kurtosis of 3.98, while the water cut has a skewness of 1.22 and a kurtosis of 0.90. It is also shown that a lot of zero production points exist in the data set, which means there are some data points related to nonproducing periods.

Regarding the skewness of fluid rate and water cut, there are two solutions: either to get rid of the outliers using some kind of outlier removal or to use the log transformation for those target variables, resulting in a normal distribution of the independent variables flow rate and water cut that will assist in enhancing the modeling results. Regarding the reported zero production points, dropping them is a must, as they are just noise data on sensors during the shut-in time. Those points will go through a preprocessing in the later step.

**Figure 4:**
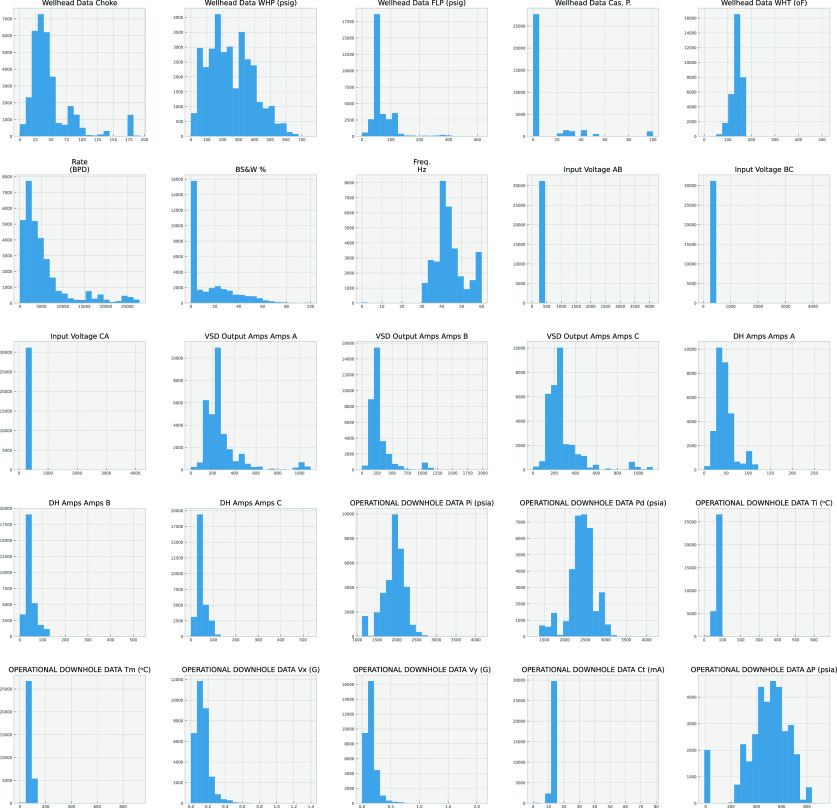
Pair plot histogram for signals.

**Figure 5:**
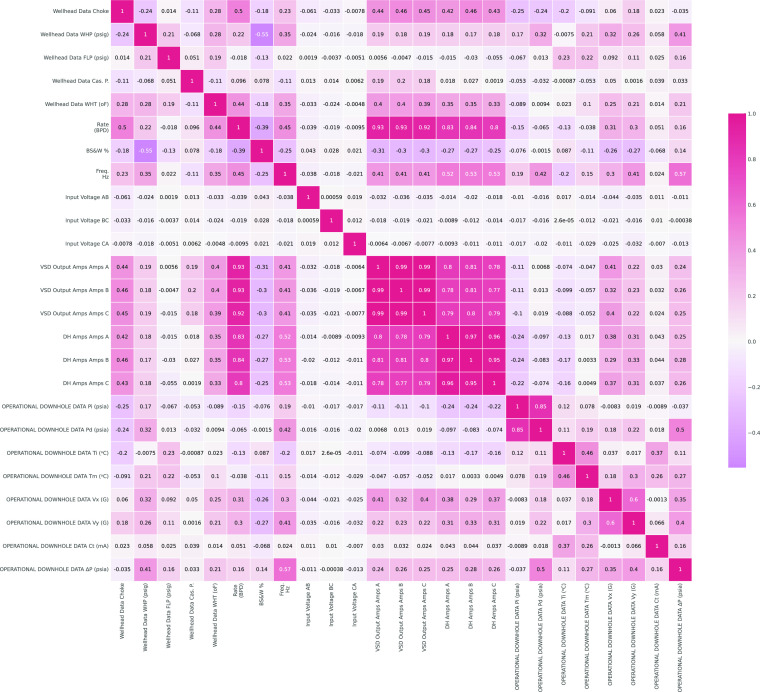
Correlation matrix for signals.

The second step is the multivariate study, in which it is assessed how the dependent variable and independent variables are related. Figures 4 and 5 show the histogram for each signal as a univariate figure and the correlation matrix for them as a multivariate study for signals. From Figure 4 it is shown that the flow rate has an approximately similar distribution with current from the variable speed drive, vibrations in (*x*, *y*), and choke opening. Also Figure 5 shows that the flow rate has a high relation with current from the variable speed drive and choke opening, while basic sediments and water are related more to wellhead pressure and a slight inverse proportionality with current and flow rate.

**Figure 6:**
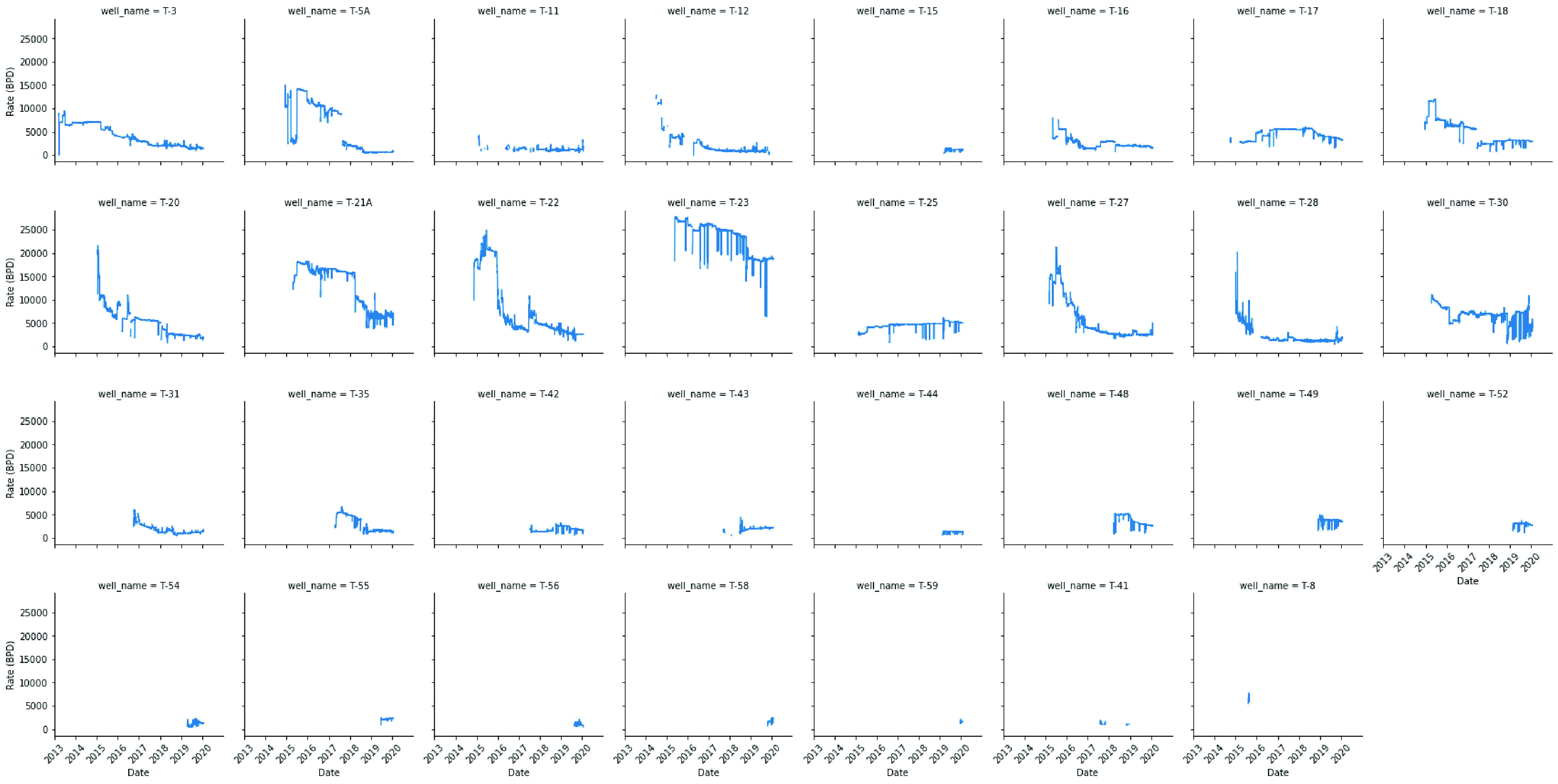
Time plot of flow rate for each well.

**Figure 7:**
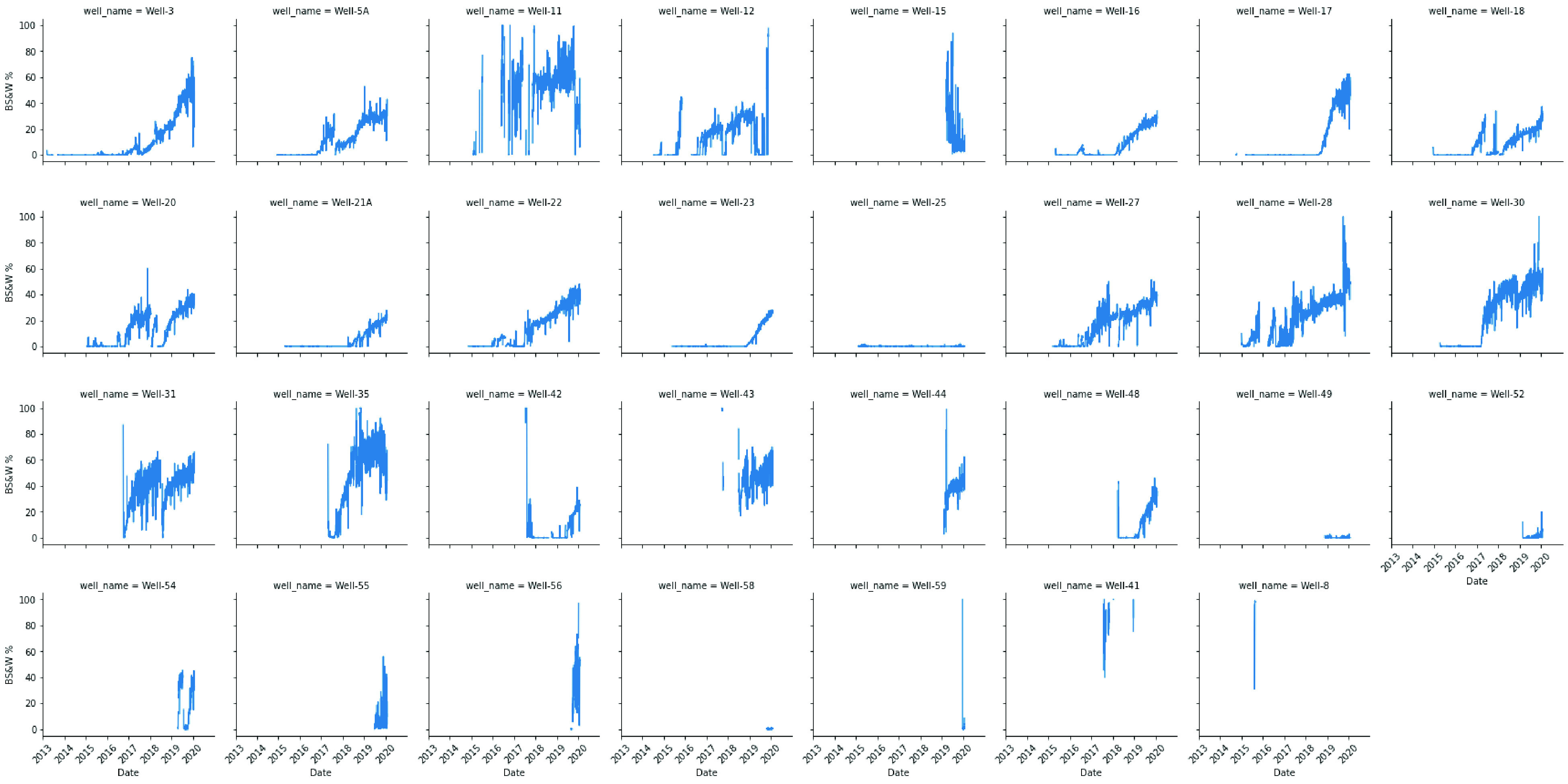
Time plot for basic sediments and water for each well.

**Figure 8:**
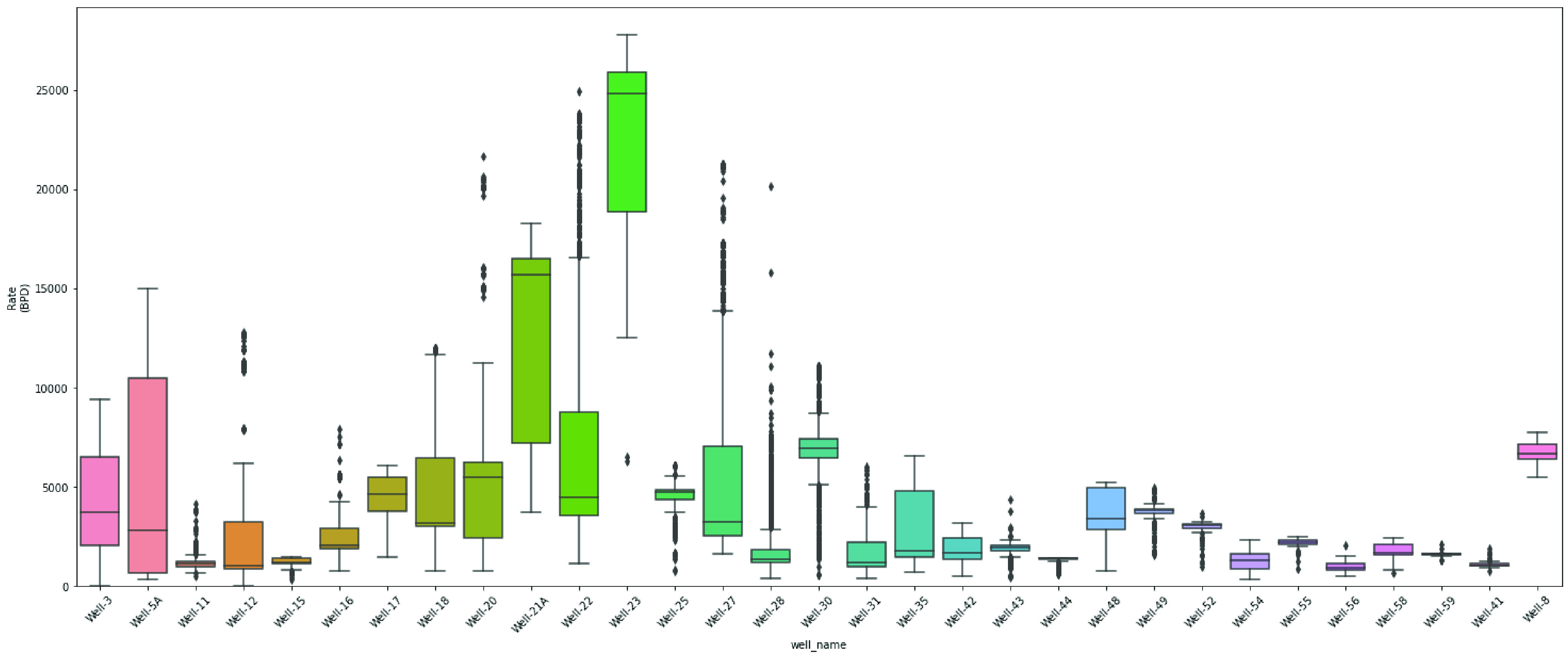
Box plot of flow rate for each well.

**Figure 9:**
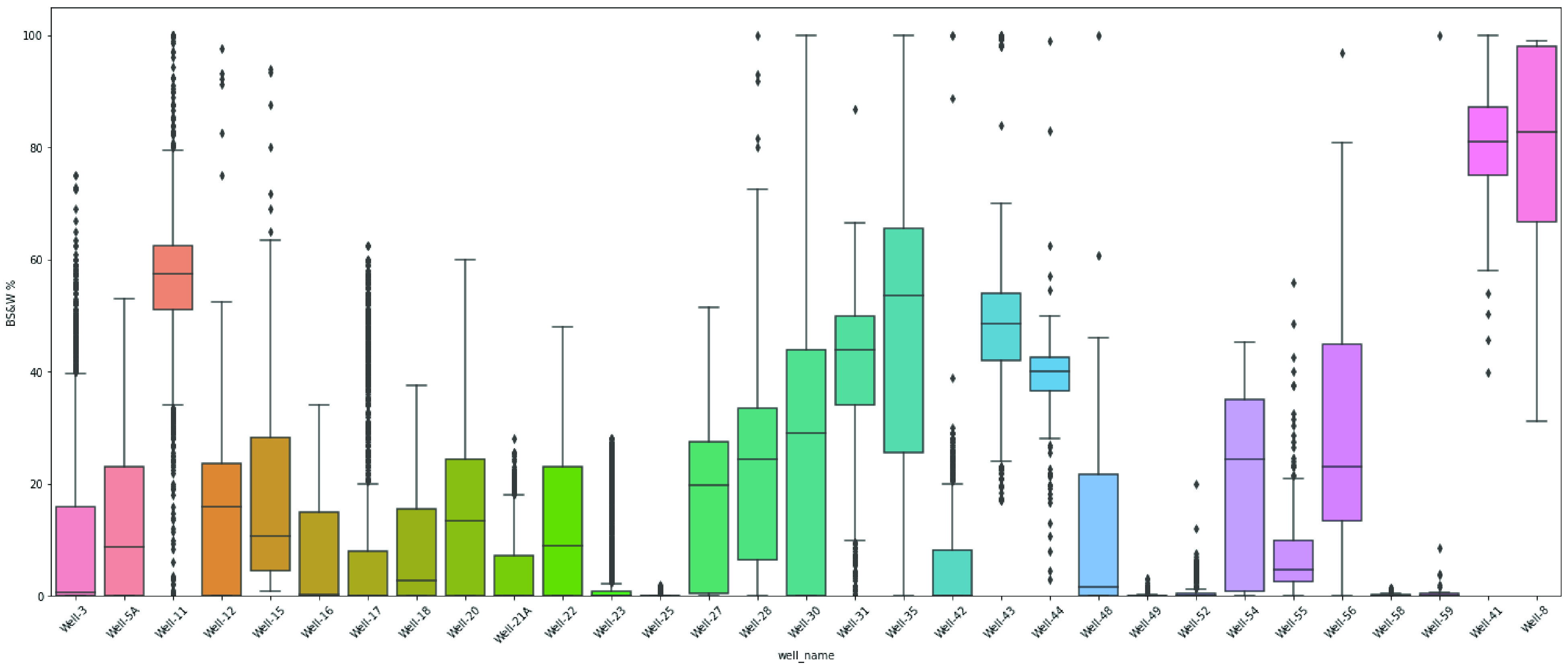
Box plot of basic sediments and water for each well.

The third study investigates the correlation between flow rate and basic sediments and water, taking into account the categorical feature of well names. Categorical features that exhibit time trends are examined in Figures 6 and 7. To demonstrate the spread of numerical data and highlight the locality, the data is grouped and shown in box plots, as depicted in Figures 8 and 9.

The missing data points are dropped for the five main signals. Those are production data because it does not make sense to have zero production reported in sensor measurements, and the remaining four signals are variable speed drive output current, pump intake pressure, frequency, and discharge pressure. The missing data for all signals are presented in Table 3. Hence, the three-phase input voltage signals (AB, CA, and BC) are dropped because, in addition to their small correlation factor with fluid rate and basic sediment and water signals that are shown in Figure 5, they also have the highest number of missing values, as shown in Table 3. Then, the motor current signal is removed because it is highly correlated with the variable speed drive output recorded current and has been considered one of the highest missing signals.

**Table 3:** Missing Data Per Signal

Reported signal	Total	Percent
Input voltage AB	2098	0.067
Input voltage CA	2098	0.067
Input voltage BC	2098	0.067
DH amps A	1469	0.047
DH amps C	1468	0.047
DH amps B	1468	0.047
*T*_*m*_ (°C)	1446	0.046
*C*_*t*_ (mA)	1444	0.046
*V*_*x*_ (G)	1443	0.046
*T*_*i*_ (°C)	1440	0.046
*V*_*y*_ (G)	1440	0.046
BS&W (%)	590	0.019
Wellhead data FLP (psig)	341	0.011
Wellhead data WHT (°F)	280	0
Wellhead data Cas. P.	12	0
*P*_*d*_ (psia)	0	0
Wellhead data choke opening	0	0
VSD output amps amps A	0	0
*P*_*i*_ (psia)	0	0
VSD output amps C	0	0
VSD output amps B	0	0
Wellhead data WHP (psig)	0	0
Frequency (Hz)	0	0
Rate (BPD)	0	0
Δ*P* (psia)	0	0

**Figure 10:**
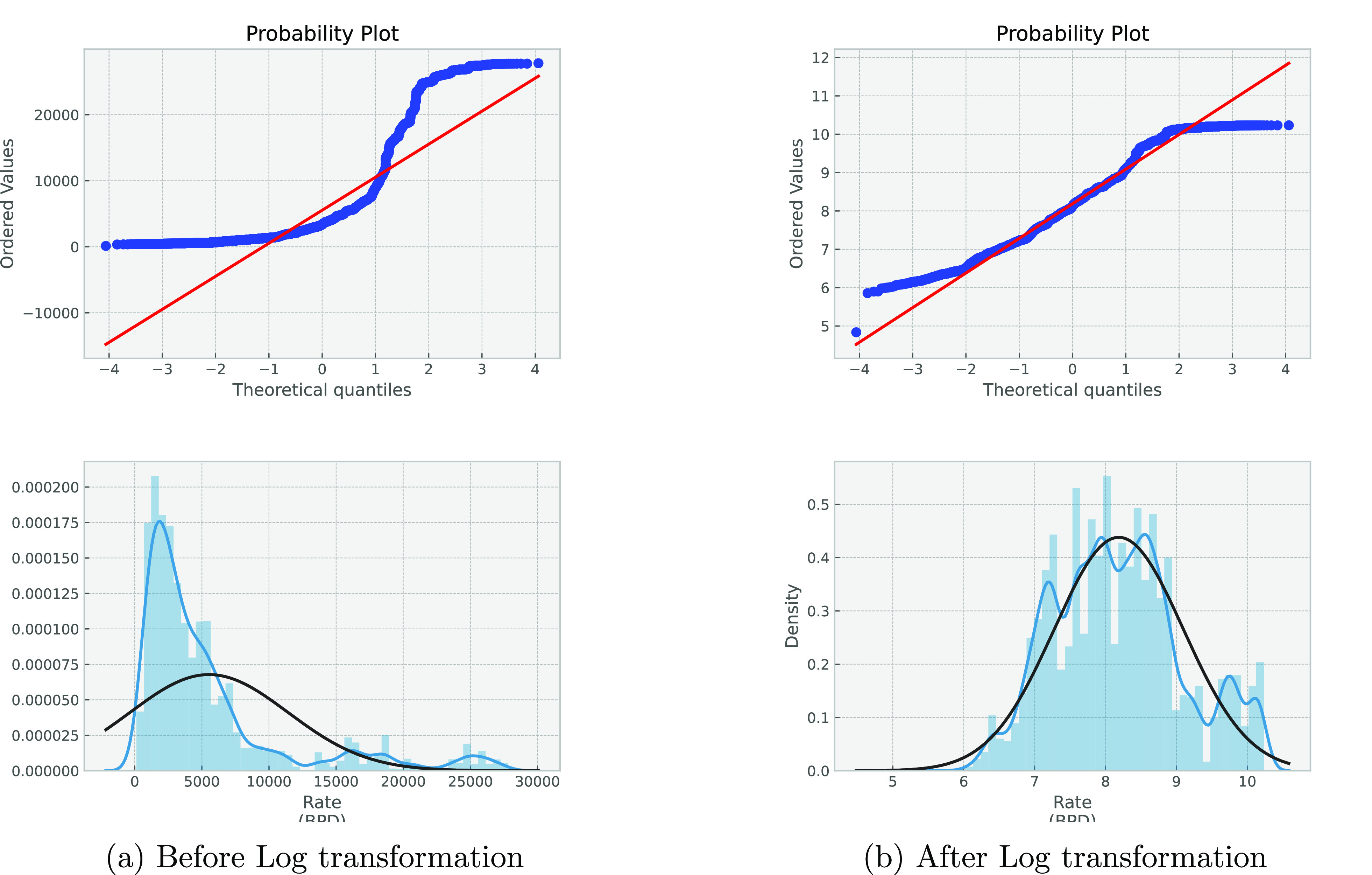
Probability plot and the distribution for flow rate.

Regarding data transformation, Figure 10 shows that the target flow rate probability density distribution has skewness from the normal distribution. Hence, the log transformation is used for the flow rate sensor measurements. The log transformation is a widely used method to address skewed data. In order to make the data as normal as possible and thus increase the validity of the associated statistical modeling, log transformation data is used instead of the Cartesian transformation. Figure 10 shows the probability plot and the distribution for flow rate before and after log transformation.

**Figure 11:**
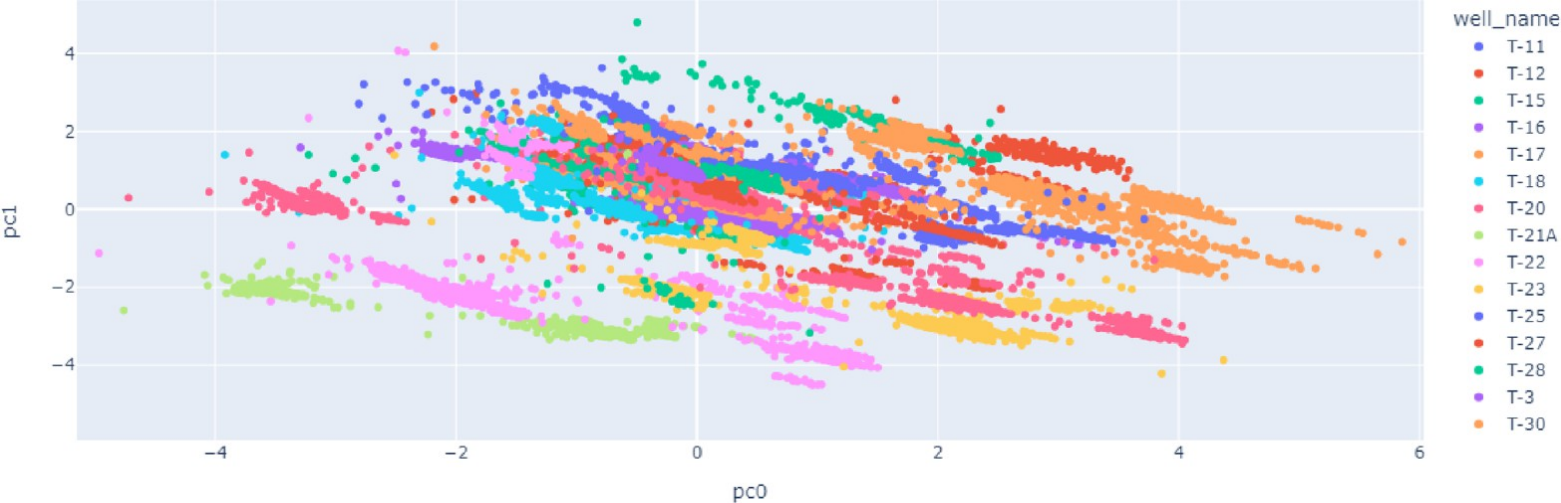
PCA of the input signals per well.

**Figure 12:**
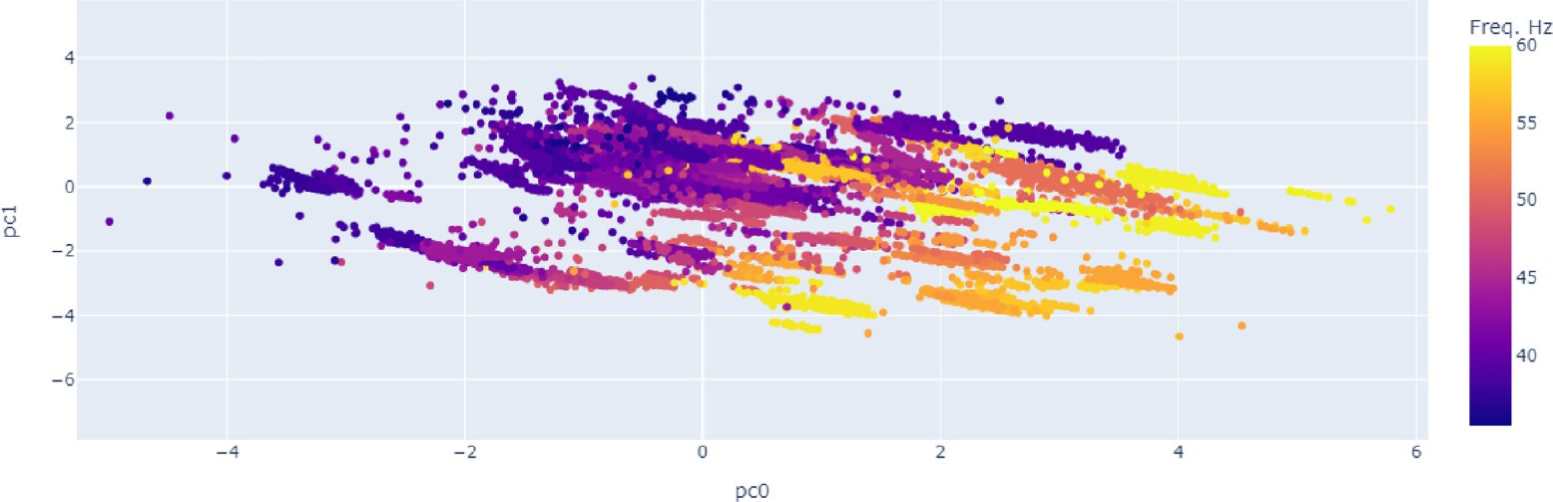
PCA of the input signals with frequency.

**Figure 13:**
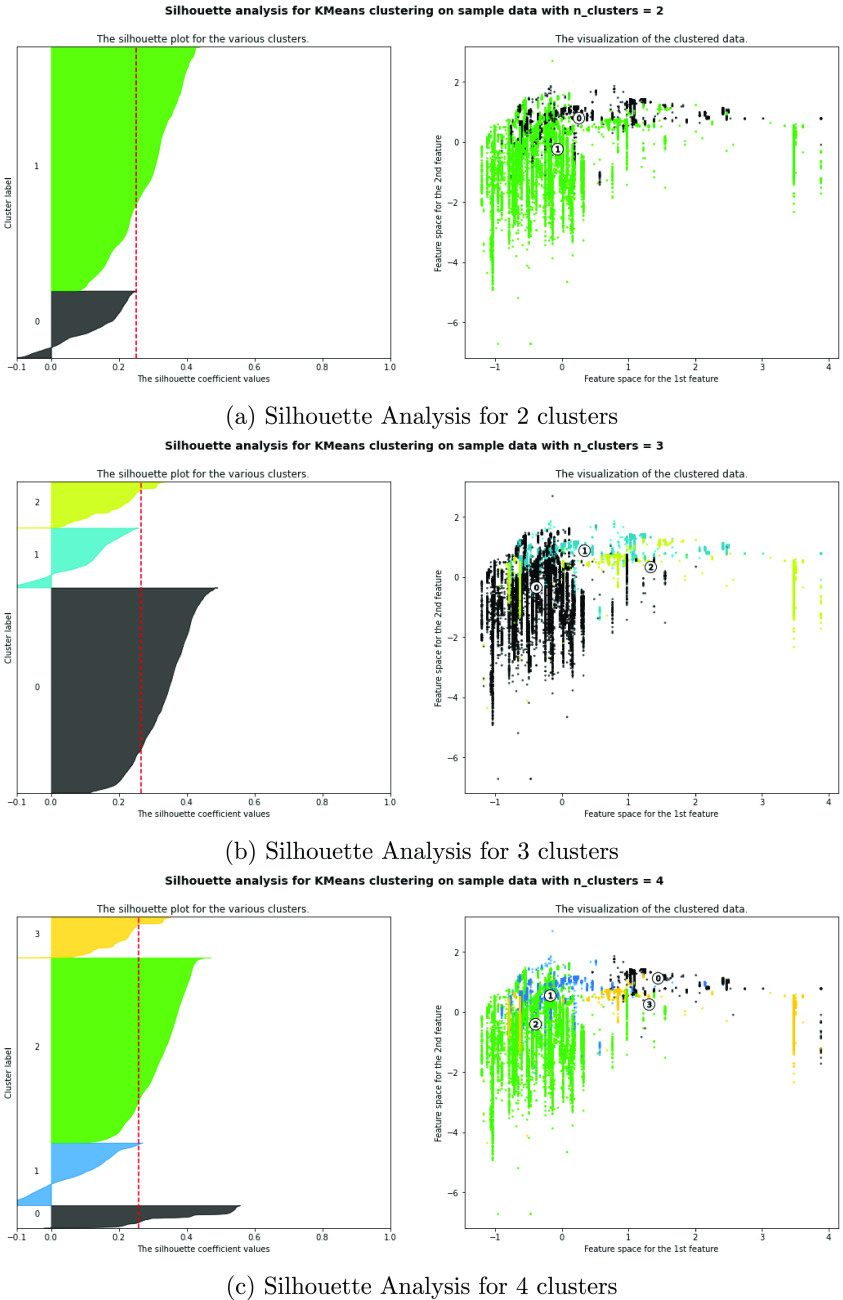

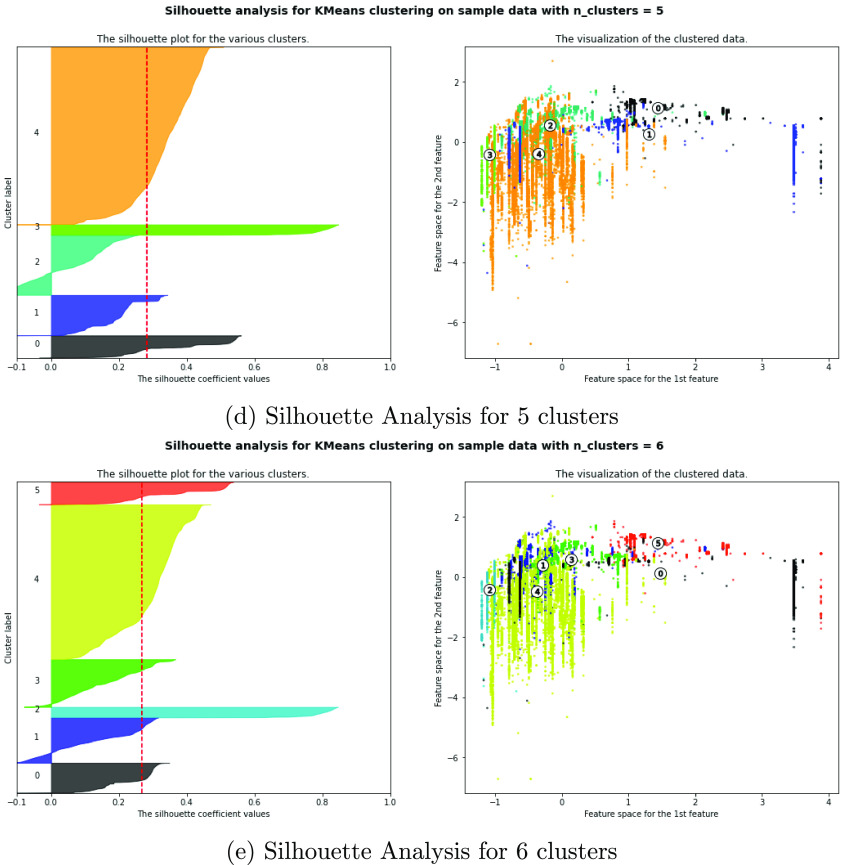
Silhouette analysis from 2 to 6 clusters.

Another important study in data exploration is the usage of unsupervised learning techniques to unleash any grouping in the input data. Hence, principal component analysis and K-means algorithms are studied. Figures 11 and 12 show the projection of the input signals on the first two principal components. There is a slight clustering per well and a slight grading of the input signals with pump frequency, which means we may have a slight improvement on modeling either by using well name or frequency as a categorical parameter. Also, K-means has been applied to the input signals to study the effect of grouping the data before modeling. Figure 13 shows the silhouette analysis of K-means clustering. Silhouette analysis can be used to study the separation distance between the resulting clusters. The silhouette plot displays a measure of how close each point in one cluster is to adjacent points in the neighboring clusters and thus provides a way to assess parameters like the number of clusters visually. This measure has a range of [−1, 1]. Silhouette coefficients near +1 indicate that the sample is far away from the neighboring clusters. A value of 0 indicates that the sample is very close to the decision boundary between two neighboring clusters, and negative values indicate that those samples might have been assigned to the wrong cluster. From Figure 13, it can be observed that the best average silhouette score is for five clusters and has a value of 0.28. Therefore, clustering is not recommended for such a case.

### Feature Permutation

A custom type of feature permutation importance model has been applied. Feature permutation is a model-agnostic method that provides insights into a machine learning model’s behavior. It estimates and ranks the importance of features based on the impact that each feature has on the trained machine learning model’s predictions.

Feature permutation measures the predictive value of a feature for our estimator. It evaluates a specific input by determining the average prediction error for all models in which this input is incorporated. The following steps summarize how we compute our custom feature permutation importance:

1. Split the data set into train and test sets.
2. Get all permutations for the input features.
3. Randomly shuffle the feature column in the given data set.
4. Train each model on the same training set.
5. Calculate the prediction error on the test data set.
6. Store the used input features with the training and testing results.
7. Repeat the preceding three steps multiple times and save the results in experiments dataframe.
8. For each input feature, the average error is reported for all models it has contributed on. Averaging mitigates the effects of random shuffling.
9. Rank the features based on the average impact each feature has on the model’s score. Features that have contributed to the smallest error models are assigned with higher importance.

Tables 4 and 5 show the signals recommended as input for flow rate and water cut, respectively, based on the feature rotation random search. In both tables, each signal is reported with the average mean absolute error (MAE) of the models in which it was used as an input. Thus, the lower the average MAE for a particular signal, the more significant its contribution is to create the best models, and vice versa.

**Table 4:** Feature Rotation for Flow Rate

Signal	Average MAE
VSD output amps (A)	0.039
Operational downhole data *P*_*d*_ (psia)	0.040
Operational downhole data *P*_*i*_ (psia)	0.041
Frequency (Hz)	0.041
Wellhead data choke	0.041
Wellhead data WHP (psig)	0.041
Wellhead data FLP (psig)	0.041
Operational downhole data *T*_*m*_ (°C)	0.041
Wellhead data Cas. P.	0.041
Operational downhole data Δ*P* (psia)	0.041
Wellhead data WHT (°F)	0.041
Operational downhole data *T*_*i*_ (°C)	0.041
Operational downhole data *V*_*y*_ (G)	0.042

**Table 5:** Feature Rotation for BS&W

Signal	Average MAE
VSD output amps A	2.16
Operational downhole data Δ*P* (psia)	2.20
Wellhead data WHP (psig)	2.21
Operational downhole data *P*_*d*_ (psia)	2.21
Wellhead data choke	2.22
Operational downhole data *P*_*i*_ (psia)	2.24
Operational downhole data *T*_*m*_ (°C)	2.31
Operational downhole data *V*_*y*_ (G)	2.31
Operational downhole data *T*_*i*_ (°C)	2.35
Wellhead data Cas. P.	2.38
Wellhead data FLP (psig)	3.40
Freq. Hz	3.41
Wellhead data WHT (°F)	4.41

## Algorithms

Usually, the ML models perform differently based on the given data. That is why there is no rule of thumb for picking a specific model for a certain task. The best model can be chosen only after the data is analyzed and the different ML models are compared. In this section, the algorithms used in this research are described. These algorithms start with symbolic regression, extreme gradient boosting (XGBoosting) and deep learning algorithms, and 1D convolutional neural network (CNN) and are used to predict flow rates and BS&W.

### Symbolic Regression

Discovering hidden relationships between variables in symbolic regression is accomplished by turning data into explicit mathematical formulas.^32,33^ The steps of a symbolic regression optimization are (i) take a data set with two or more columns; (ii) propose formulas for one of the columns as a function of the other ones; and (iii) evaluate the errors and keep track of the recordholders.

Intrinsic stochasticity is involved: random formulas must be sequentially tried. Because too many possible formulas exist, a clever algorithm must be used. It is a powerful tool to help understand the underlying dynamics of an observed phenomenon. Instead of fitting numbers to some presumed model, as the classic regression does, this method optimizes the functional form itself of the relationship between the variables. This can often help to discover nonlinear correlations between the variables that regular regression models just could not predict.

However, care must be taken to avoid overfit models, where spurious correlations between the variables are given too much merit, leading to good fits with little predictive value. To avoid this risk, the use of cross-validation is recommended. The method takes care of turning its intrinsic distributions and correlations into meaningful models.^34,35^

### XGBoosting

A decision-tree-based ensemble of predictive models is created using the machine learning approach known as gradient boosting for classification and regression issues. The residuals or errors of the prior model are computed at each iteration. These residuals will be predicted by the subsequent model added to the decision-tree ensemble, which will then put them all together to provide the final forecast.^36−39^

Starting with a single root, the procedures for building an XGBoost model are followed (containing all of the training samples). Second, all characteristics and the values that correspond to them are iterated through. Finally, each potential split loss reduction is assessed. The objective function (loss function and regularization) that must be reduced at each iteration is indicated by eqs 1 and 2.
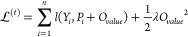
1
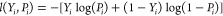
2where *Y*_*i*_ is the true value required to be predicted of the *i*^th^ instance, *P*_*i*_ is the prediction of the *i*^th^ instance, *l*(*Y*_*i*_, *P*_*i*_) is the loss function for a typical classification problem, and *O*_*value*_ is the output of the new tree.

 is the regularization term.

Chen and Guestrin clarified that it is impossible to optimize the XGBoost objective function in Euclidean space using conventional optimization techniques.^40^ Therefore, the second-order Taylor approximation is utilized to convert this objective function to the Euclidean domain, allowing for the employment of conventional optimization techniques. The loss function’s Taylor approximation is shown in eq 3,
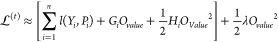
3where *G*_*i*_ is the gradient and calculated by  and *H*_*i*_ is the Hessian, calculated by .

The derivative of the goal function is obtained by subtracting the constant component and obtaining eqs 5 and 6.
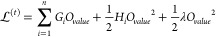
4
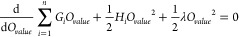
5
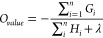
6

By merging eq 6 with the first and second derivatives of the classification loss functions *G*_*i*_ and *H*_*i*_, the similarity equation is derived. The similarity score is calculated as follows in eq 7:
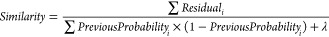
7

A leaf of the tree is used to determine the similarity score. To divide the tree into additional leaves, different criteria are utilized. For each fresh leaf, the similarity score is computed, and subsequently the alleged gain as shown in eq 8 is computed.
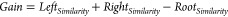
8

As the tree continues to grow, thresholds are set again until higher gain thresholds are reached. A certain quantity of residuals must be present in each leaf for the tree to stop developing. Calculating a parameter called “cover” yields this result. Cover is defined as the similarity score’s denominator less lambda. Based on Chen et al. (2006), “It is described as the sum of second order gradient of training data classified to the leaf, if it is square loss, this simply corresponds to the number of instances in that branch. Deeper in the tree a node is, lower this metric will be”.^40^

The process is carried out during boosting in such a way that trees are built one after the other. Each tree updates the residual mistakes while simultaneously reducing the error of its predecessor and learning from it. As a consequence, each tree that grows in the sequence will gain knowledge from an updated version of the residuals.

In addition, the base learners’ predictive capability, which is just marginally better than random guessing, makes them poor at boosting. Nevertheless, each of these underachievers provides some crucial data for prediction. By merging these weak learners into a single strong learner by boosting, a significant learning effect is achieved that lowers both the bias and the variance.

### Deep Learning

Deep learning methods have lately demonstrated considerable advantages in automatically extracting and learning multivariate data characteristics for enormous data and high computing performance. Particularly, feature extraction of time series data has been applied to the recurrent neural network (RNN) and its several enhanced models. In this article, CNN and LSTM are just two examples of the multivariate prediction issues offered for a generic framework. Before the LSTM network layer, CNN is used to extract the horizontal correlations between multidimensional variables, and LSTM is used to learn the temporal relationships of these features and make predictions based on them.^41−45^

**Figure 14:**
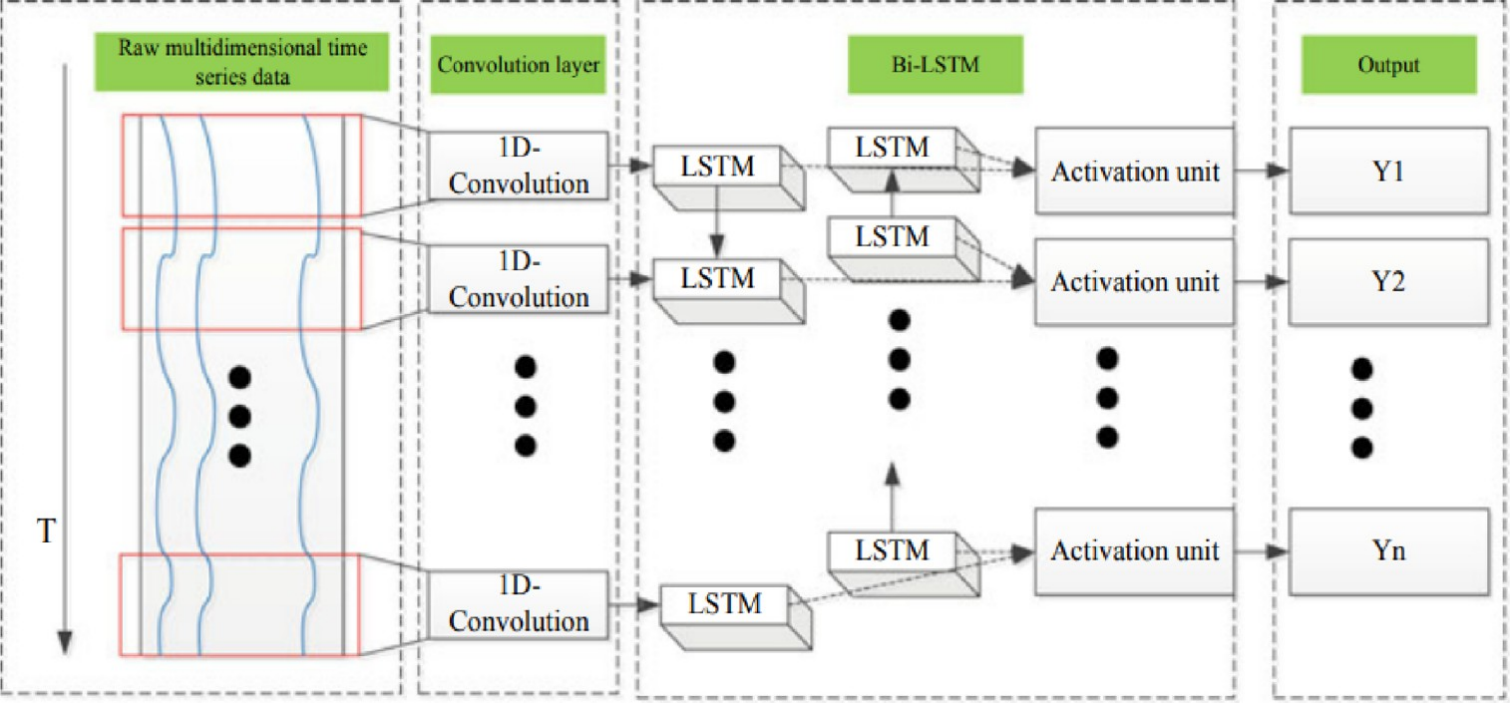
Model framework.

There are two primary components in this model. As seen in Figure 14, CNN is used to extract the lateral characteristics of multidimensional data, while LSTM is used to recover the temporal features.

Such a network architecture brings together the advantages of time series processing by using LSTM and signal processing for the data that comes from pump sensors by using a 1D convolutional network.

#### Convolutional Neural Networks

In general, conventional neural networks are designed to operate exclusively on 2D data such as images and videos. In a conventional multilayer perceptron (MLP), each hidden neuron contains scalar weights, input, and output. However, due to the 2D nature of images, each neuron in a CNN contains 2D planes for weights, which is known as the kernel, and input and output, which is known as the feature map.

CNN has three main types of layers: a convolutional layer, a pooling layer, and, finally, a fully connected layer. A convolutional layer is the main building block of a CNN. It contains a set of filters (or kernels), the parameters of which are to be learned throughout the training. The size of the filters is usually smaller than the actual image. Each filter convolves with the image and creates an activation map. For convolution, the filter slides across the height and width of the image, and the dot product between every element of the filter and the input is calculated at every spatial position. The second type of layer is a pooling layer. It is a downsampling layer, or in other words, a dimensionality reduction layer, that is used to lower the computational complexity of the network by reducing the eigenvectors of the convolutional layer output while also extracting the key features and performing feature compression.^46^ Lastly, the pixel values of the input image or signals are not directly connected to the output layer in partially connected layers. However, in the fully connected layer, each node in the output layer connects directly to a node in the previous layer. Therefore, the fully connected layer performs the task of classification based on the features extracted through the previous layers and their different filters.^47−51^

**Figure 15:**
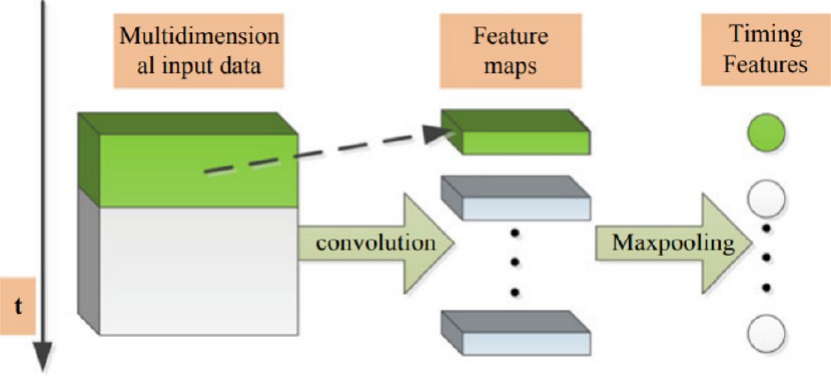
One-dimensional convolution.

CNN is considered a powerful tool for image and signal processing because it fuses the feature extraction and feature classification processes into a single learning body. What makes them also attractive for sensors’ signal processing is that they are immune to small transformations in the input data, including translation, scaling, skewing, and distortion. Consequently, a lot of recent applications of time series data analysis and signal processing apply 1D convolutional neural networks.^54−61^ It can be considered a modified version of 2D CNNs. 1D CNNs could be more advantageous over 2D CNNs in dealing with 1D signals for many reasons. The low computational complexity of 1D CNN, of course, would come first. Also, as a general observation from recent studies, most of the 1D CNN applications have used compact architectures (with 1–2 hidden CNN layers), whereas almost all 2D CNN applications have used deep architectures. Obviously, networks with shallow architectures are much easier to train and implement. To conclude, compact 1D CNNs are well-suited for real-time and low-cost applications due to their low computational requirements.^52−64^Figure 15 depicts a one-dimensional CNN model.

In each CNN layer, 1D forward propagation (1D-FP) is expressed in eq 9,
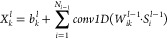
9where *X*_*k*_^*l*^ is defined as the input, *b*_*k*_^*l*^ is defined as the bias of the *K*^th^ neuron at layer l, *S*_*i*_^*l*–1^ is the output of the *i*^th^ neuron at layer (*l* – 1) to the *k*^th^ neuron at layer l, and *conv1D* is used to perform “invalid” 1D convolution without zero padding. Therefore, the dimension of the input array, *X*_*k*_^*l*^, is less than the dimension of the output arrays, *S*_*i*_^*l*–1^. The intermediate output, *y*_*k*_^*l*^, can be expressed by passing the input *X*_*k*_^*l*^ through the activation function, *f*(.), as follows: .

The main idea behind the back-propagation algorithm is to compute the gradient of the error with respect to the weights of the network and then use this gradient to update the weights in a way that reduces the error. In the back-propagation algorithm, the error is propagated backward through the network, from the output layer to the input layer. This is done using the chain rule of calculus to compute the gradient of the error with respect to the weights. The gradient is then used to update the weights in a way that reduces the error. In eq 10, *t*^*p*^ represents the target output for input *p*, and  represents the output of the network for input *p*. The sum in the equation computes the mean-squared error between the target output and the actual output of the network.
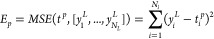
10

During the back-propagation algorithm, the error at the output layer is first computed using eq 10 and then propagated backward through the network. At each layer, the error is split and distributed to the neurons based on their contribution to the error. The weights of the connections between neurons are then adjusted in a way that reduces the error.

#### Long Short-Term Memory Algorithm

LSTM is one of the most intricate subfields in deep learning. It is a difficult concept to comprehend. The long short-term memory network adds cells to the information storage module, which realizes long-term memory of the sequence data and solves the problem of gradient disappearance and gradient explosion in RNN networks.

**Figure 16:**
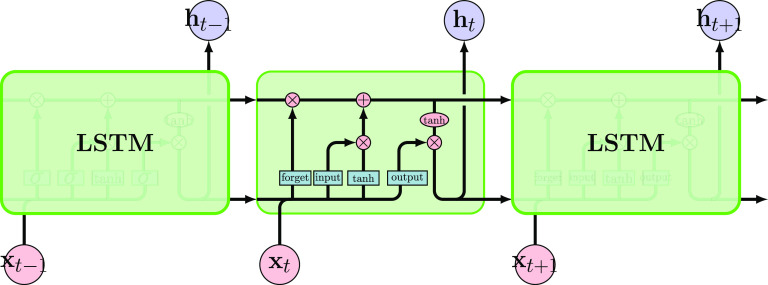
Structure of LSTM cells.

In the LSTM, the internal states of the unique unit, known as the memory cell, are used by the architecture to guarantee consistent error flow. Updating the state of the cell through three gating units is the key to LSTM, as shown in Figure 16. The three gating units have different calculation methods and functions. A forget gate, an input gate, and an output gate make up a typical LSTM unit.^65−68^

In a forget gate, the sigmoid function generates a value between 0 and 1, which is the first step to indicate how much knowledge of the prior hidden state and the present input it should keep. In the input gate, the following action has two components. The input gate chooses the new data to be stored in the memory cell first. A tanh layer then creates a vector of new candidate values that will be added to the state. In the output gate, applying the sigmoid function to the prior hidden state and current input and multiplying it by the tanh function applied to the new memory cell will provide values between −1 and 1 for the memory cell’s output.^66,69−71^ The equations for the forget, input, and output gates in LSTM are presented in eqs 11–13,
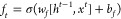
11
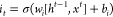
12
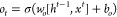
13where *f*_*t*_ represents the forget gate, *i*_*t*_ represents the input gate, *o*_*t*_ represents the output gate, σ represents the sigmoid function, *W*_*x*_ is the weight for the respective gate (*x*) neurons, *h*^*t*–1^ is the output of the previous LSTM block (at timestamp *t* – 1), *x*^*t*^ is the input at the current timestamp, and *B*_*o*_ represents the biases for the respective gates (*x*).

The equations for the cell state, candidate cell state, and final output are presented in the following eqs 14–16,
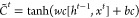
14
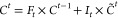
15
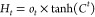
16where *C*^*t*^ is the cell state (memory) at timestamp (*t*) and  represents a candidate for the cell state at timestamp (*t*).

The gate units of the bidirectional LSTM are identical to the LSTM. Bi-LSTM is a combination of a forward LSTM and a backward LSTM, which means that one time step of data is input simultaneously in both forward and reverse directions. Although Bi-LSTM needs to train more generations to converge to stability, it also has higher precision because it receives more input information, which is the reason why the Bi-LSTM is chosen as the prediction model to be implemented.

## Experiments

In this section, we explore the proposed machine learning models through experiments aimed at tuning their hyperparameters. The input variables utilized in the liquid rate production models include variables such as variable speed drive current, choke opening percent, pump frequency, intake pressure and temperature, wellhead pressure and temperature, and pump discharge pressure, which are chosen due to their relevance to liquid production in oil wells. Similarly, the recommended input variables for the basic sediment and water cut prediction model include wellhead pressure and temperature, choke opening, pump frequency, variable speed drive current, pump intake pressure and temperature, flow line and casing pressure, pump vibrations, and motor temperature. We conduct experimentation with different hyperparameters, such as learning rates, regularization parameters, and activation functions, in order to optimize the performance of the models. It is important to note that hyperparameter tuning is a crucial step in developing robust and effective machine learning models.

For symbolic regression, all candidate solutions are represented by regular functions, whose structure is determined from building blocks defined by sets of input variables, constants, and function symbols. For the present regression, all symbol sets are considered functions containing addition, subtraction, multiplication, division, power, minimum, constant, integer constant, and input variable operations (+, −, *, /, *pow*, min, and *c*, respectively).

The fitness function that associates a numerical fitness value to each candidate solution and defines the problem to be solved by the genetic programming is the mean absolute error (MAE) of the normalized production rate and BS&W approximation against the normalized measured value from both signals. While a formal hyperparameter tuning still has to be conducted, a preliminary tuning using a heuristic for the extrapolated trajectory against the true model was conducted. The selected hyperparameters are shown in Table 6.

**Table 6:** Symbolic Regression Hyperparameter

Hyperparameter	Value
Binary operators	+, −, *, /, *pow*
Unary operators	exp, sin, cos, sqrt
Loss	MAE
Number of populations	5000
Number of generations	50

Regarding the XGBoost algorithm, hyperparameters are divided into three categories. These categories are known as general parameters, booster parameters, and learning task parameters. General hyperparameters define the type of algorithm to be either linear or tree-based, the verbosity to print results, and the number of threads to run on. Booster parameters include the main tuned parameters for the algorithms such as learning rate, the minimum sum of weights of all observations required in an internal node in the tree, and the learning parameters to specify the minimum loss reduction required to make a split. Table 7 shows ranges that are used for hyperparameter tuning. A random search has been used for 1000 iterations, and the best set of such parameters is used to build the proposed model.

**Table 7:** Hyperparameter Tuning

Parameter	Reference to	Sampling type	Range
max_depth	Control of overfitting, higher depth facilitates such that the model learns relations that are specific to a particular sample	Suggest integer value	2, 10
min_child_weight	Minimum sum of weights defined for all observations required in a child	Log uniform	1e–10, 1e10
colsample_bytree	Subsample ratio of columns when constructing each tree	Uniform	0, 1
learning_rate	Overfitting prevention through step size shrinkage in updates	Uniform	0, 0.1
Gamma	Specification of the minimum loss reduction required to make a split	Suggest integer value	0, 5

For deep learning applications, there is a large number of hyperparameters. Some are the variables that determine the network structure (e.g., number of hidden units and size and type of the network layers). On the other hand, there are variables that determine how the network is trained (e.g., learning rate and batch size).

In this study, we followed the recommendations of Kiranyaz et al. (2020)^72^ on the structure. The configuration of the 1D CNN-LSTM used in all experiments has 2 hidden convolutional layers, and they are followed by the average pooling layer that down-samples the input representation by taking the average value over a window of 5. The 1D CNNs have 32 and 16 neurons on the first and second hidden convolutional layers, respectively, while the two LSTM layers that followed that average pooling have 32 neuron followed by dropout of 0.25. Then, two dense layers are added, 10 neurons on the hidden dense layer. Finally, the output layer size is 2 which is the BS&W and liquid rate.

For all experiments, the maximum number of back-propagation iterations is set to 150, and another stopping criterion is the minimum train Huber error level, which is set to 1% to prevent overfitting. Therefore, the training will terminate if either of the criteria is met. Initially, the learning factor, *e*, is set at 10^–3^, and global learning rate adaptation is performed during each BP iteration, as follows: if the train MSE decreases in the current iteration, *e* is slightly increased by 5%; otherwise, it is reduced by 30%, for the next epoch. In addition, all models are trained using the Adam algorithm, which optimizes a predetermined loss function with the goal of obtaining the final model. Regarding the loss function, we have tried the mean squared error and Huber function. However, the reported model uses only the Huber function, eq 17, as it is found to have more stable models than mean squared error. That was also expected. The Huber function is reported in the literature to be used in robust regressions because it is less sensitive to outliers in the data than the squared error loss.
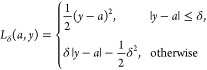
17

## Results and Discussion

Detailed exploratory data analysis, outlier removal, data transformation, and feature ranking have been done. Models have been built using symbolic regression, XGBoosting, and Conv1D with LSTM algorithms. In this section, the models created by the aforementioned algorithms for prediction on sensors’ data measurements have been evaluated, and the most appropriate models for further analysis on model-agnostic evaluation metrics have been selected. Finally, the predicted values coming from the proposed models are shown, and they are compared to the actual values belonging to various wells of data.

### Comparison of the Applied Algorithms

In this study, data sets were utilized to implement various algorithms. These data sets were divided into two sets: the training set and the testing set. The time series split technique was used to create these sets, wherein the future timeline data were excluded from the testing set. All testing metrics were calculated based on the testing set.

The training set was used for training and validation purposes and was subjected to a 10-fold cross-validation method. This allowed for a comprehensive evaluation of the models’ performance, ensuring that the models were tested on different subsets of the training data. The utilization of these techniques enabled a thorough analysis of the algorithms implemented in the study, and the obtained results were reported based on the testing metrics.

When comparing different models, it is important to use objective metrics that can quantify their performance. Three commonly used metrics are mean absolute error (MAE), mean squared error (MSE), and R-squared. These metrics are used to evaluate the accuracy of a model in predicting outcomes on a test data set, which is a set of data that was not used during the model development process. In addition to the aforementioned three metrics, it is common to use a cross plot to compare the predicted values of each model with the actual values in the test data set. In the following paragraphs, we will provide a more detailed explanation of testing metrics and cross plots to further enhance the understanding of their significance in model evaluation.

Mean absolute error (MAE) is a metric that calculates the average of the absolute differences between the actual and predicted values. It measures the average magnitude of the errors in the predictions and provides a measure of how close the predictions are to the actual values. A lower MAE indicates better accuracy of the model.

Mean squared error (MSE) is another metric that calculates the average of the squared differences between the actual and predicted values. This metric is similar to MAE, but it penalizes larger errors more heavily than smaller errors. A lower MSE also indicates better accuracy of the model.

R-squared (R^2^) is a metric that measures how well the predicted values fit the actual values. It is a statistical measure of the proportion of the variance in the dependent variable that is predictable from the independent variable(s). R^2^ ranges from 0 to 1, with a higher value indicating a better fit. A value of 1 means that the model perfectly predicts the outcome, while a value of 0 means that the model does not explain any of the variation in the outcome.

These metrics are important for scientific evaluation of the effectiveness of a model because they provide objective measures of the model’s performance. By comparing the MAE, MSE, and R-squared of different models, scientists can determine which model is better suited for predicting outcomes in their particular domain.

Finally, a cross-plot is a graphical representation of the relationship between two variables. In scientific research, cross-plots are often used to assess the performance of different models by comparing the predicted values to the actual values. By comparing the distribution of the points on the graph to the 45° line, researchers can assess the accuracy of each model. A model with all points lying on the 45° line would have perfect accuracy, while a model with points scattered randomly around the graph would have poor accuracy.

Tables 8 and 9 present a comparison of the models built using symbolic regression, extreme gradient boosting, and a convolutional-LSTM neural network to predict liquid rate and basic sediment and water (BS&W) in oil wells. The convolutional-LSTM models were found to provide the best results for both liquid rate and BS&W prediction. However, the XGBoosting algorithm also performed well in both cases, particularly for BS&W prediction.

**Table 8:** Models Comparison for Liquid Rate Prediction

Metric	Symbolic regression	XGBoosting	CONV1D-LSTM
MAE	0.551	0.127	0.113
R-squared	0.44	0.92	0.95
MSE	0.243	0.046	0.041

**Table 9:** Models Comparison for Basic Sediments and Water Prediction

Metric	Symbolic regression	XGBoosting	CONV1D-LSTM
MAE	0.41	0.031	0.036
R-squared		0.908	0.905
MSE	0.21	0.005	0.004

The convolutional-LSTM model outperformed the other two models in liquid rate prediction, with a mean absolute error (MAE) of 0.113, the highest R-squared value of 0.95, and a mean squared error (MSE) of 0.041. The XGBoosting model also showed good results, with an MAE of 0.127, an R-squared value of 0.92, and an MSE of 0.046. However, the symbolic regression model had the highest MAE of 0.551, the lowest R-squared value of 0.44, and the highest MSE of 0.243.

For BS&W prediction, both the convolutional-LSTM and XGBoosting models produced good results, with MAE values of 0.036 and 0.031, R-squared values of 0.905 and 0.908, and MSE values of 0.004 and 0.005, respectively. The symbolic regression model had the highest MAE of 0.41 and the highest MSE of 0.21. The negative predictions produced by the symbolic regression model for some data points made it impossible to calculate the R-squared value for this model.

**Figure 17:**
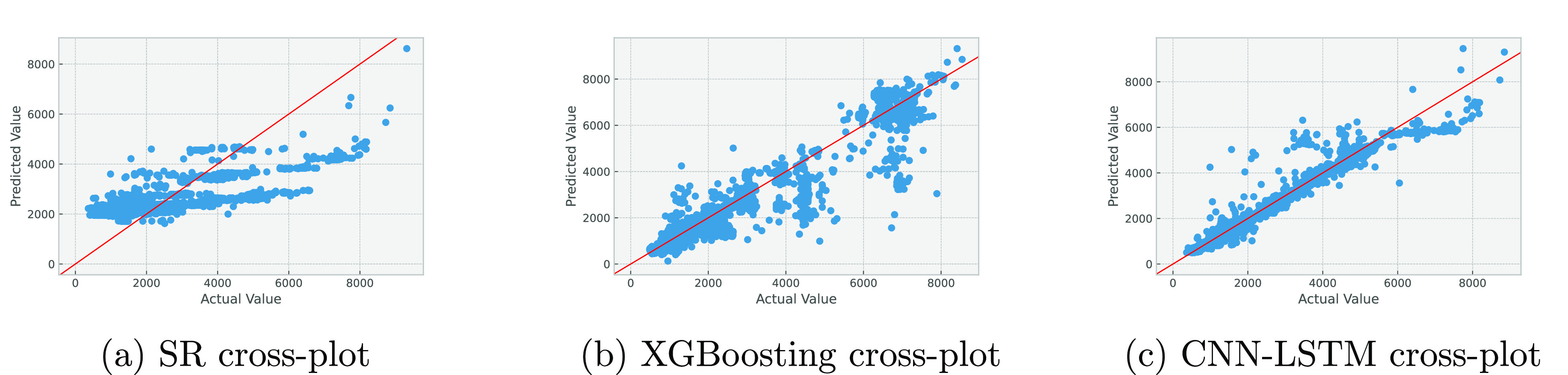
Cross-plots for liquid production prediction using various algorithms.

**Figure 18:**
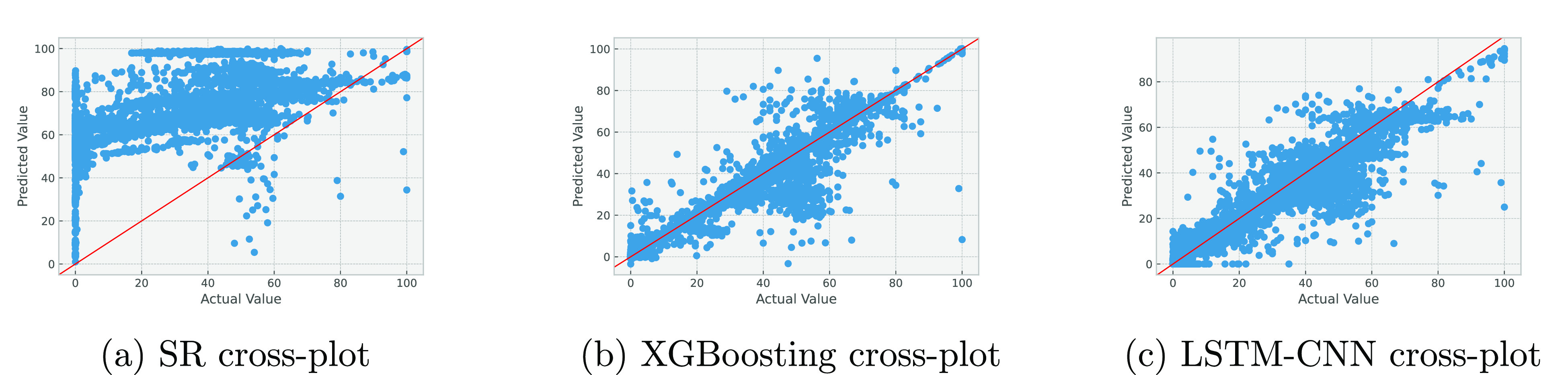
Cross-plots for basic sediments and water cut prediction using various algorithms.

Figures 17 and 18 show the cross-plots of predicted versus actual values for the three algorithms used for liquid rate and BS&W prediction, respectively. As we have mentioned, these plots provide a visual representation of the performance of each model and illustrate the closeness of the predicted values to the actual values.

### Deep Learning Results

**Figure 19:**
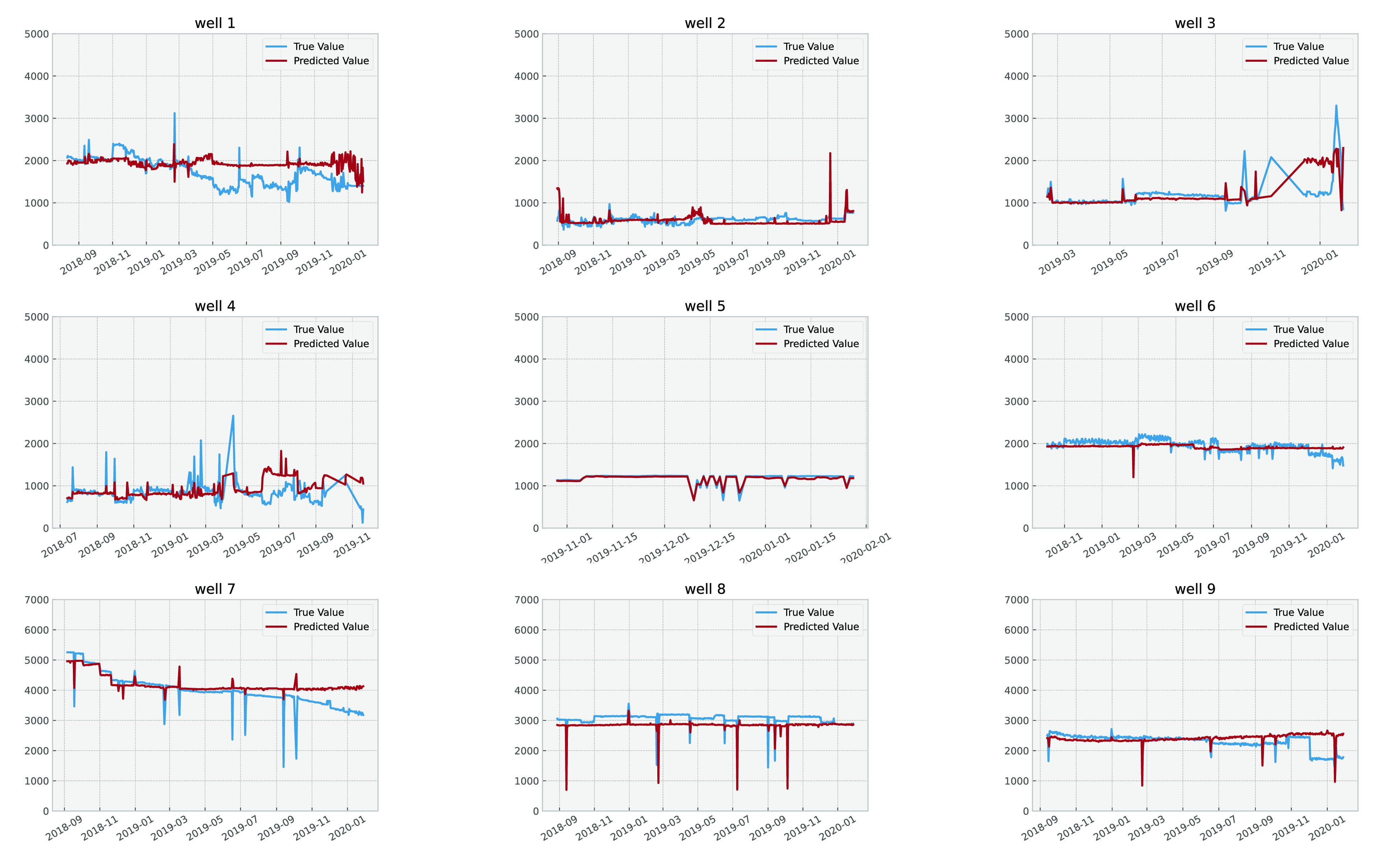
Liquid rate production per well, including predicted values and actual values.

**Figure 20:**
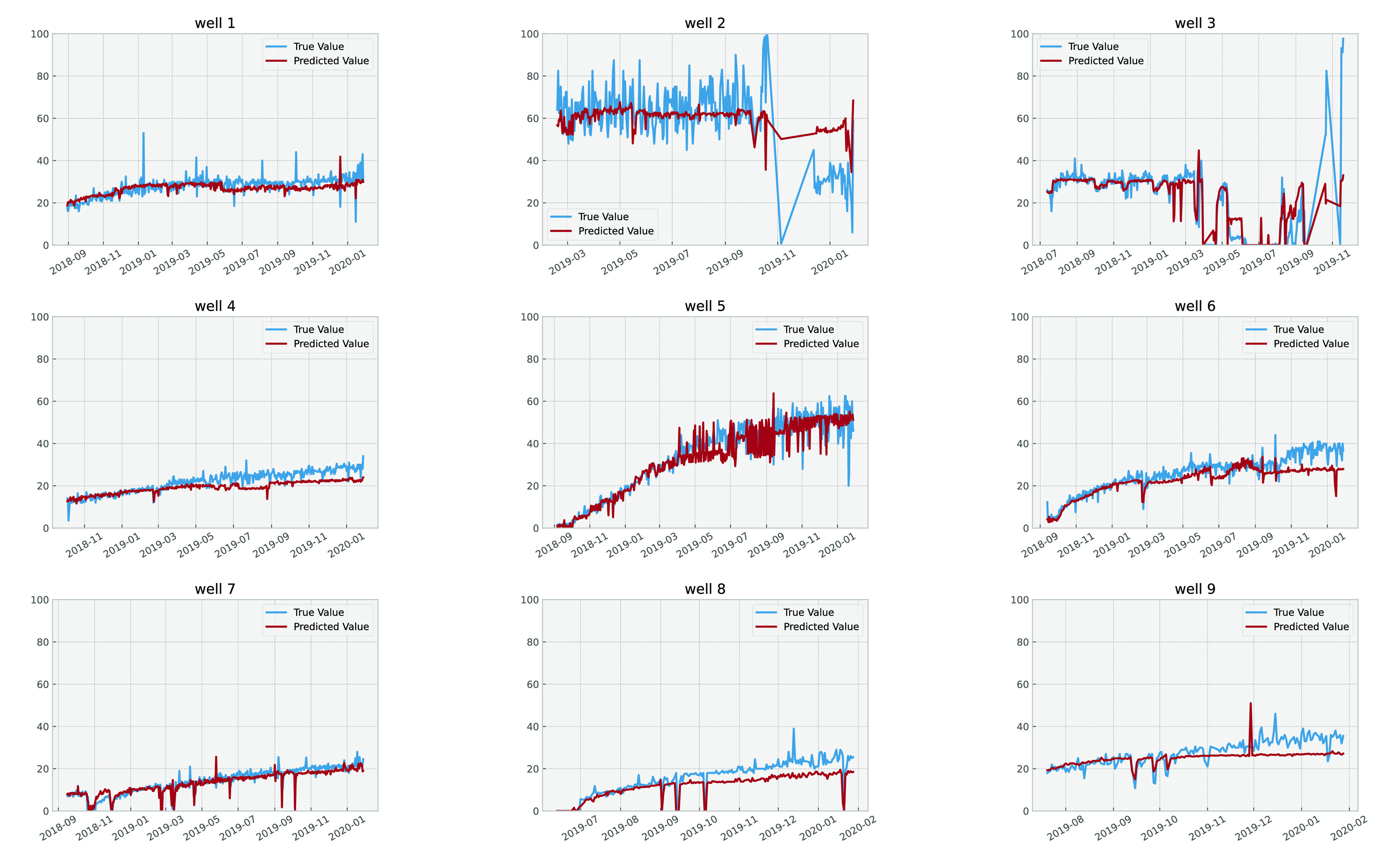
Basic sediments and water cut percent per well, including predicted values and actual values.

Among the models that were constructed, those using deep learning showed the best results on the test set. Figures 19 and 20 demonstrate the effectiveness of the model by quantitatively evaluating it on data sets that were not used during the training process. These data sets include the last 30% of nine wells, which is approximately the data from the last two years.

For liquid rate prediction, the predicted values were quite accurate for more stable wells (e.g., wells 2, 5, and 6). However, for wells like 1 and 3, the results had higher errors. Similar outcomes were observed regarding basic sediments and water percent prediction, with good results for most of the wells, except for those showing a drastic change in BS&W percent (e.g., well 2 as shown in Figure 20). This could be due to such wells having unstable conditions and requiring a lot of interventions or because the model’s predictability is not sufficient for such wells.

## Conclusions and Recommendations

This research presents a novel approach to data-driven modeling of multiphase flow rate using the ESP sensors data set. The study includes a comprehensive exploratory data analysis and features prioritization experiments, which lead to the identification of the most significant parameters for flow rate prediction. The proposed framework for modeling includes symbolic regression, XGBoost, and deep learning methods, specifically a CNN-LSTM pipeline, to predict liquid rate and water cut in real time. The model’s ability to provide instantaneous predictions using only nine variables demonstrates its feasibility for this specific application.

The study’s novel approach is rooted in its comprehensive and systematic analysis of the ESP sensors data set, which includes a custom feature permutation method to estimate and rank feature importance. The proposed approach identified and ranked the nine most significant parameters for flow rate prediction, providing valuable insights into the underlying data patterns. These insights can inform future modeling studies and optimize the selection of relevant parameters for flow rate prediction.

The framework of modeling from more interpretable and less complex models, such as symbolic regression, to more complex and less interpretable models, such as deep learning methods, is another unique contribution of this study. This approach allows for the identification of the most significant parameters through a more interpretable and transparent model and the subsequent use of more complex models to improve predictive performance. This methodology can be applied to other flow rate prediction problems and serves as a valuable contribution to the field of data-driven modeling.

Additionally, the study highlights the importance of evaluating the predictability of data-driven models using an independent testing data set, a step that is often overlooked in the existing literature. The use of an independent testing data set ensures that the model’s predictive performance is generalizable to new data and is not solely optimized for the training data set. This approach enhances the reliability and accuracy of the proposed model and provides a valuable contribution to the field of data-driven modeling.

The implementation of the proposed CNN-LSTM pipeline for time series analysis of sensor data also provides a valuable contribution to the field of data-driven modeling. The 1D-CNN layers were able to extract features and create informative representations of time series automatically. They are highly noise-resistant models, and they are able to extract very informative deep features, which are independent of time. Likewise, LSTM networks are really effective and reliable at extracting patterns in input feature space, where the input data spans over long sequences. The unique gated LSTM’s architecture has the ability to manipulate its memory state in such a way that long-term memory can be stored. This unique architecture is particularly suitable for time series analysis of sensor data, making it a valuable contribution to the field of data-driven modeling.

Finally, the study highlights the importance of high-quality input data for reliable predictions. The proposed approach emphasizes the need for a comprehensive exploratory data analysis to identify and mitigate data quality issues, such as missing data and outliers. The study’s findings emphasize the importance of high-quality input data for reliable predictions and provide valuable insights into the data-driven modeling of ESP wells’ virtual flow metering. Overall, this research presents a valuable contribution to the field of data-driven modeling for ESP wells’ virtual flow metering.

## List of Abbreviations

**1D****/2D**: One/two-dimensional
**1D-FP**: One-dimensional forward propagation
**BS****&W**: Basic sediment and water
**CFD**: Computational fluid dynamics
**CNN**: Convolutional neural networks
**EDA**: Exploratory data analysis
**ESP**: Electric submersible pump
**FPI**: Failure prediction index
**LSTM**: Long short-term memory algorithm
**MAE**: Mean absolute error
**ML**: Machine learning
**MPFMs**: Multiphase flowmeters
**MSE**: Mean squared error
**PCA**: Principal component analysis
***P_d_***: Discharge pressure
***P*_*i*_**: Intake pressure
**RNN**: Recurrent neural network
**RRL**: Remaining run life
**SSA**: Singular spectrum analysis
**VFM**: Virtual flow metering
**VSD**: Variable speed drive
**WHP**: Wellhead pressure
**WHT**: Wellhead temperature
